# Relationship Among Green Human Resource Management, Green Knowledge Sharing, Green Commitment, and Green Behavior: A Moderated Mediation Model

**DOI:** 10.3389/fpsyg.2022.924492

**Published:** 2022-06-30

**Authors:** Kalimullah Khan, Muhammad Shahid Shams, Qaisar Khan, Sher Akbar, Murtaza Masud Niazi

**Affiliations:** ^1^Department of Business Administration, Kardan University, Kabul, Afghanistan; ^2^Department of Arts and Media, Foundation University, Islamabad, Pakistan; ^3^Department of Management Sciences, Comsats University, Islamabad, Pakistan

**Keywords:** GHRM, green behaviors, green knowledge sharing, green commitment, organization psychology

## Abstract

This study aims to examine the underlying mechanism of the relationship between perceived green human resource management (GHRM) and perceived employee green behavior (EGB). By drawing on attitude and social exchange theories, we examined green commitment (GC) as a mediator and green knowledge sharing (GKS) as a moderator of the GHRM–EGB relationship. The study employs partial least square structural equation modeling (PLS-SEM) to analyze 329 responses. Data were collected in two time lags. The empirical results confirmed that GC mediates the relationship between GHRM and EGB. However, the study results found that GKS moderated the indirect influence of GHRM on green behavior *via* GC. This research signifies the effect of GHRM, GKS, GC, and green behavior on organizations’ sustainability and environmental management. Despite the emerging literature on the significance of green practices in organizations for environmental management, no study has examined the moderating role of GKS on the indirect effect of GHRM on green behavior *via* mediating role of GC. This study offers valuable insight into environmental management in organizations through green practices and green behavior.

## Introduction

Environmental performance is the main challenge for current organizations for their sustainability (Comin et al., 2019; Pham et al., 2019; Hameed et al., 2020). As environmental management has become the main concern for governments, authorities, and regulators encourage organizations toward green products and services (Comin et al., 2019; Cop et al., 2020; Yong et al., 2020a; Rubel et al., 2021). By considering this emerging development in environmentalism, organizations are shifting their traditional models into green models for environmentally improved performance (Mousa and Othman, 2020; Rubel et al., 2021). In search of green models, the literature suggests the role of green human resource management (GHRM) practices in an organization (Singh et al., 2020). GHRM promotes employee environmental performance for environmental sustainability (such as minimizing paper use, reducing waste, and promoting water recycling for sanitation purposes; Singh et al., 2020; Rubel et al., 2021). Literature also suggests that to boost the environmental performance of an organization, it is prudent to focus on developing the environmental skills, attitudes, and behaviors of employees (Saeed et al., 2019; Yong et al., 2020b; Islam et al., 2021b). Based on behavioral research on HRM, GHRM can affect employees’ work attitudes and behaviors through social and psychological processes (e.g., Comin et al., 2019; Pham et al., 2019; Singh et al., 2020). Prior studies of HRM reported relationships between GHRM practices and employee work outcomes, such as employee pro-environmental behavior (Rubel et al., 2021), psychological green climate (Dumont et al., 2016; Islam et al., 2020), social proof (Shen et al., 2018), green employee empowerment (Hameed et al., 2020; Islam et al., 2021a), pro-environmental psychological capital (Saeed et al., 2019), GHRM, and employee green behavior (EGB; Fawehinmi et al., 2020). However, research on environmental performance for organizations’ sustainability through employee’s green behavior is still in the emerging stage and calls for further research to explore more social and psychological mechanisms to explain GHRM–EGB relationships (Pham et al., 2019; Rubel et al., 2021; Bhatti et al., 2022). Heeding this literature call, this study investigated GHRM and employees’ green behavior through green commitment (GC) and green knowledge sharing (GKS) as an underlying mechanism in textile sector of Pakistan.

The textile industry is Pakistan’s most important branch of industry. Some 15 million people (around 40% of the workforce) are employed in this sector. Textile companies are vast enterprises with offices, residential halls, and event halls that consume much energy and other resources based on the human activities (de Souza Freitas et al., 2011; Gomez and Yin, 2019). The carbon emissions issue is more prominent in the manufacturing sector with large populations encompassing great physical space, whose layout also includes the use of vehicles (Abdul-Azeez, 2021; Islam et al., 2021b). Furthermore, considering the vast space and the population in large companies, the textile sector produces a large chunk of waste including plastics, papers, and e-waste (Tangwanichagapong et al., 2017; Vrontis et al., 2021), which could contaminate the water supply and environment if disposed of improperly. Therefore, for the successful implementation of an environmental management system, EGB is paramount (Dumont et al., 2017; Fawehinmi et al., 2020; Islam et al., 2020; Singh et al., 2020). Studies have shown that the participation of employees is key to a successful EMS in an organization (Mazzi et al., 2016; Rubel and Jones, 2016; Yong et al., 2020a; Islam et al., 2021a). Furthermore, EGB allows an organization to achieve a competitive advantage in terms of its environmental performance (Kim et al., 2019; Fawehinmi et al., 2020). Therefore, EGB carries more importance for the environmental performance of organizations for their sustainability and for sustainable society. Therefore, based on strong theoretical underpinnings and prior studies’ recommendations on the need to explore underlying mechanisms of GHRM-performance outcomes, this study investigates the moderating role of GKS on the indirect influence of GHRM on green behavior *via* GC by employing the theoretical underpinnings of attitude theory (Bull, 1951) and social learning theory (Bandura, 1977; Bandura and Hall, 2018). Based on attitude theory (Bull, 1951), we suggest that GHRM affects green behavior through GC. Based on social learning theory (Bandura, 1977), we further suggest that employees who adopt organizations’ GHRM initiatives and share green knowledge will also influence other members and can become a source of inspiration and can help the rest of the employees to adopt the same practices to become part of the members follow green practices and share green knowledge in the working relationship. This consequently has a positive impact on employees’ learning with their environmental commitment and green behavior. Thus, attitude theory supports the mediating role of GC, while social learning theory supports GKS as a moderating variable in this study (refer to the “Theory and Hypotheses” section).

In summary, our study contributes to the empirical literature on environmental management and GHRM in various ways. First, our study tests the mediating role of GC as an underlying mechanism between GHRM and green behavior through the lens of attitude theory (Bull, 1951). Second, our study contributes to GKS as a moderating variable between GHRM and GC. Third, our study contributes to GKS as a moderating variable in the indirect relationship between GHRM and green behavior *via* GC by applying social learning theory (Bandura, 1977) in a South Asian context (Pakistan).

The remainder of this study discusses the theoretical background and hypotheses, research method, and study results. Finally, we close our study by discussing the results, implications, limitations, and directions for future research.

## Theory and Hypotheses

Attitude theory (Bull, 1951) and social learning theory have been used to explain the theoretical underpinnings of this study. Attitude theory (Bull, 1951) supports the mediating role of GC, while social learning theory supports GKS as a moderating variable. Attitude theory signifies the importance of employees’ positive behaviors like affective commitment (Babakus et al., 2003) in the mediation of employees’ positive evaluations of management practices and their positive responses. Based on attitude theory, it is argued that organizations adopting and implementing GHRM practices make employees more committed to their environment (Pham et al., 2019). Prominent scholars (Roscoe et al., 2019; Tuan, 2021) have proposed the importance of GC as a mediator linking GHRM to the various employee and organizational outcomes. Therefore, GC can be viewed as an employee commitment to environmental issues (Paillé et al., 2014), serving as an intermediary link between GHRM and green behavior. Therefore, an organization’s GHRM practices affect employees’ GC s, affecting their green behaviors.

**FIGURE 1:**
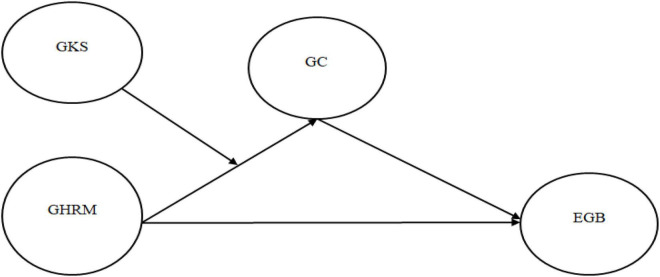
Research model. GKS, green knowledge sharing; GHRM, green human resource management; GC, green commitment; and EGB, employee green behavior.

Furthermore, social learning theory states that individuals learn new behaviors by observing and imitating others. This theory describes learning in the social context as a cognitive process that can be realized by instructions or observations even if there is no direct reinforcement. On the basis of social learning theory, we suggest that employees who adopt organizations’ GHRM initiatives and share green knowledge will also influence other members, become a source of inspiration, and can help the rest of the employees to adopt the same practices to become part of the members follow green practices in the working relationship. This consequently has a positive influence on employees’ learning with their environmental commitment and green performance. Prior studies have reported the influence of GHRM on employees’ green behavior *via* the indirect effect of GC (Farrukh et al., 2019; Ansari et al., 2021). However, we believe this indirect relationship can be strengthened by sharing employees’ green knowledge. It is proposed that employees supporting this culture possibly be inspired further to increase the employees’ GC and green service behavior. This facilitates the linkage of GHRM, GKS, and green behavior *via* GC as depicted in Figure 1.

### Green Human Resource Management and Green Commitment

The GHRM refers to the management of human resources while considering the environmentally sustainable performance of organizations (Renwick et al., 2016; Afsar et al., 2018; Islam et al., 2020; Yong et al., 2020a). This includes employee hiring, staffing, training, and performance assessments based on green standards. In the GHRM framework, employees’ performance is measured and rewards are given based on the green targets’ achievements (Renwick et al., 2016; Teixeira and Queirós, 2016; Yusliza et al., 2017). GHRM seeks to mobilize and ensure the employees’ participation in the level of greater green competence that brings the cost to the minimum level wherever possible, like virtual interviews, online training, job sharing, teleconferencing, and building energy efficient offices (Rubel et al., 2020). GHRM is applied when the HRM actions are aligned with environmental management (Rubel et al., 2020). This strategy depicts that the organization is moving its business approach toward green initiatives (Renwick et al., 2016). Literature suggests that HRM influences the performance of organizations through employees’ attitudes and behaviors (Alfes et al., 2013; Norton et al., 2014). So, GHRM also impacts green behavior within the employee workplace (Luu, 2019; Fawehinmi et al., 2020). This helps improve the employees’ green values and this will consequently lead to the employees’ environmental awareness and behavior (Renwick et al., 2016). Likewise, employees’ job descriptions and responsibilities can be associated with environmental responsibilities. Similarly, employees’ performance, promotion, and rewards may be based on an employee’s green contribution in the organization, and this may inspire other staff to realize their green intentions by accomplishing green targets (Renwick et al., 2016). It is argued that workers’ views and thinking on why institutions and organizations accept various HRM policies describe how those actions will affect the employee’s behavior (Rubel et al., 2018). In the same way, a structured connected set of GHRM measures indicates to employees the GC of organizations and, subsequently, it is expected of them to exhibit behaviors in line with the organization policies. Literature also suggests that GHRM stimulates employees’ green behavior, attitude, and commitment to the environment (Saeed et al., 2019; Fawehinmi et al., 2020; Islam et al., 2021b). An employee’s GC is an individual’s feeling and attachment toward an organization’s environmental management. GC is an outcome of GHRM representing employees’ attitudes and his/her organizational values and his/her efforts toward the environmental performance of organizations (Pham et al., 2019; Chaudhary, 2020). GHRM positively influences the GC of the employees. Previous studies like Pham et al. (2019), Rubel et al. (2018), Ren et al. (2018), Fawehinmi et al. (2020), Ansari et al. (2021), and Islam et al. (2021b) have confirmed GHRM’s positive relationship with individual-level green constructs. Therefore, we believe that if employees view GHRM as positive, it will enhance their GC and lead us to the following hypothesis:

**H1**: Green human resource management has positive effect on employees’ GC.

### Green Commitment and Green Behavior

Employee commitment is the psychological state of employees that demonstrates the extent of employees’ attachment with their organization (O’Reilly and Chatman, 1986). Literature suggests that employee commitment directs his/her behavior (Pham et al., 2019; Ansari et al., 2021). GC depends on the employee’s psychological attachment, his/her consideration of organizational goals and values, and his/her sense of environmental responsibility (Kim et al., 2019). Therefore, GC is considered employee affiliation, engagement, identification, involvement, and concern for the workplace ecological environment (Afsar et al., 2020). If the workers have a weak commitment and less passion for the environment, they are conceivably less worried about the ecological issues during their daily activities in the workplace. Individual involvement and affection positively affect employees’ behavior (Ren et al., 2018; Afsar and Umrani, 2020). So, employee GC is related to green behavior such as energy management, switching off extra lights, recycling, and overall concern for the ecological issues in the workplace (Guerci et al., 2016; Cheema et al., 2019). In line with this, past studies have confirmed that emotional attachment with commitment leads to pro-environment behavior, and when there is a higher level of GC, then there is greater green service behavior (Afsar and Umrani, 2020; Cop et al., 2020). Hence, employee GC leads to employees’ green behavior. Based on the literature mentioned above, we developed the second hypothesis of our study as follows:

**H2**: Employee GC has positive effect on employees’ green behavior.

### Green Commitment as Mediator

Prior literature suggests that HRM not only affects the workers’ attitudes and behaviors directly but there are also psychological mechanisms that influence employee’s behaviors (Jiang et al., 2012; Boxall et al., 2016; Islam et al., 2020). Literature also suggests that these psychological mechanisms (e.g., GC, psychological green climate, and employees’ green engagement) may enhance work performance (Shen et al., 2018; Ansari et al., 2021; Islam et al., 2021b). We argue that employee GC conceivably mediates GHRM and performance outcomes. Prior research shows that GHRM affects employee outcomes (Ren et al., 2018; Pham et al., 2019; Saeed et al., 2019). Likewise, previous studies also show the influence of GC on green behavior (Rubel et al., 2018; Saeed et al., 2019; Afsar and Umrani, 2020; Ansari et al., 2021). Moreover Bull’s (1951) attitude theory also shows the importance of positive affective responses of workers, such as affective commitment, in mediating workers’ positive and constructive assessment of management practices and workers’ positive behavior (Babakus et al., 2003). Along the same vein, previous studies have shown consistent findings between GHRM and EGB (Cheema et al., 2019; Fawehinmi et al., 2020; Ansari et al., 2021). Furthermore, Rubel et al. (2021) in their recent research also suggested GC as an underlying mechanism of the GHRM–EGB relationship. Thus, literature prompted this study to investigate the relationship between GHRM and employee’s green behavior *via* GC. Thus, a GC may be seen as an employee’s willingness and commitment toward environmental management to exhibit green behavior (Paillé et al., 2014). Thus, it leads to the following hypothesis:

**H3**: Green commitment positively mediate relationship between GHRM and EGB.

### Green Knowledge Sharing as Moderator

Green knowledge sharing is the extent of sharing green knowledge by organizations members to improve organizational environmental performance (Lin and Chen, 2017). Prior studies acknowledge the significant role of knowledge management in the workplace (Lopes et al., 2017; Dezi et al., 2019). It is known that knowledge management influences many performance outcomes (Bhatti et al., 2022). However, the main component of knowledge management is knowledge sharing (Rubel et al., 2018). Previous studies have examined knowledge sharing at the individual and organization levels (Vrontis et al., 2021; Bhatti et al., 2022). Individually, employees can create “collaborative” knowledge by sharing their knowledge with other employees (Teh and Yong, 2011; Jabbour and de Sousa Jabbour, 2016; Song et al., 2020). Green knowledge sharing plays a significant role in the sustainable competitive advantage of the organizations (Norton et al., 2014; Gope et al., 2018; Song et al., 2020). Therefore, good green knowledge management leads to improved knowledge of environmental management in organizations (Lin and Chen, 2017). Previous studies like Ren et al. (2018), Pham et al. (2019), Islam et al. (2021b), and Rubel et al. (2021) have confirmed GHRM’s positive relationship with employees’ commitment and green practices. We propose that the influence of GHRM on GC is potentially moderated by GKS. We believe that a high level of GKS in organizations can enhance the positive relationship between GHRM and employee GC. Previous research has shown that knowledge sharing moderates employee trust and commitment, employee behavior and virtual team effectiveness (Shateri and Hayat, 2020), job satisfaction, workplace friendship, and service innovation (Song et al., 2015; Okoe et al., 2018). Furthermore, in human resource management research, knowledge sharing has been identified as a significant moderator in different relationships, such as the relationship between organizational culture and job satisfaction (Tang et al., 2018), high-commitment work systems, and employee service behaviors relationships (Rubel et al., 2018: Gilal et al., 2019) and the relationship between human resource management practices and corporate entrepreneurship (Mustafa et al., 2018). Similarly, Salopek and Dixon (2000) believed that knowledge sharing transfers information and knowledge to others to create learning opportunities and encourage others to learn. In any event, the key to knowledge always lies in individuals. The “people” in an organization will be the key factor in knowledge sharing. As per social learning theory (Bandura, 1977), employees who adopt organizations’ GHRM initiatives and share green knowledge will also influence other members and can become a source of inspiration and can help the rest of the employees to adopt the same practices to become part of the members follow green practices and share green knowledge in the working relationship. This consequently has a positive impact on employees’ learning with their environmental commitment and green behavior. Based on social learning theory, this study proposes the following hypotheses 4 and 5:

**H4:** The positive relationship between GHRM and employee GC will be strengthened when GKS is high.

### Moderated Mediation

H3 and H4 conceptually supported GC as a mediator and GKS as a moderator, where GKS increases the indirect relationship between GHRM and green behavior *via* GC. This integrative relationship is supported by attitude theory (Bull) and social learning theory (Bandura, 1977). Hypotheses 3 and 4 justify moderation-mediation integrated relationships. Furthermore, employing attitude theory, GC is a psychological and social mechanism that bridges the relationship of GHRM with green behavior. Specifically, we suggest that the more there is a high level of GKS, the more it will strengthen (as the social learning theory suggests) the indirect relationship of GC between GHRM and green behavior. Accordingly, we proposed the fifth hypothesis of our study as follows:

**H5**: Knowledge sharing moderates the indirect relationship between GHRM and green behavior *via* GC such that the indirect relationship will be stronger when knowledge sharing is high.

## Research Method

### Research Design, Sample, and Data Collection

Based on the positivist paradigm, we employed a deductive approach and quantitative methodology by applying the convenience sampling technique of non-probability sampling approach (Anderson and Gerbing, 1988; Sekaran, 2009; Creswell, 2013). We employed cross-sectional research design in a non-contrived setting for data collection in the textile sector of Pakistan. We contacted the top 25 companies in the textile industry through e-mails and direct calls for an appointment and management approval to collect data from individuals. Thus, this study is based on an individual unit of analysis. The data were collected in two time lag for a total duration of 44 days (February 04 to March 20, 2022) to avoid common method bias (CMB) issues (Podsakoff et al., 2003). A unique identifier was assigned to each questionnaire to match the participants’ responses at time one (T1) and time two (T2). In T1, a questionnaire consisted of demographic information and items on the GHRM and GKS, while in T2, the questionnaire consisted of items on GC and green behavior. We distributed 675 questionnaires in T1 and got back a response of 465 respondents. In T2, questionnaires were distributed among those who responded in T1, and 427 responded. In total, 35 questionnaires were not correctly filled out and removed, and the final useable sample was 392. The response rate in this study was 58.07%.

Moreover, we used the G-power software of Faul et al. (2009) by selecting a medium effect size of 0.15, a statistical power of 0.80, and five numbers of predictors. We got a minimum sample size of 92 subjects. Thus, minimum sample requirements are fulfilled in this study.

### Measures

#### Green Human Resource Management

We adopted six items of GHRM from Dumont et al. (2017). All the variables in this study are measured with a five-point Likert scale where 1 = strongly disagree and 5 = strongly agree. The Cronbach’s alpha value for this scale was 0.916, as validated by Islam et al. (2021b) and Malik et al. (2021) in the context of Pakistan.

#### Employee Green Behavior and Green Commitment

Bissing-Olson et al.’s (2013) six items were adopted to measure employees’ green behavior, while Raineri and Paillé’s (2016) eight items were employed to measure GC.

#### Green Knowledge Sharing

Finally, we developed five items from Wong (2013) to measure GKS. All the variables’ items’ validity ranged from 0.77 to 0.91 and were acceptable based on a threshold of 0.7 (Hair et al., 2017).

## Results

### Respondents’ Profile

Respondents’ profiles are presented in Table 1, showing the sample’s demographic characteristics. To ensure the representation of the sample and homogeneity of variance, we applied Leven’s test in SPSS based on the early and late responses. Late responses were considered after a one-time reminder for their response. A Leven statistic was found to be 0.027, *p* > 0.871. Based on this statistic, equal variances are assumed, and it was concluded that homogeneity of variance is not an issue, and the sample represents the population of the study.

**TABLE 1:** Respondents’ profile.

Characteristics	Responses (*N* = 329)
***Gender***	
Male	243
Female	86
**Age (in years)**	
20–30 years	90
31–40 years	91
41–45 years	124
51–60 years	25
**Experience**	
1–5	76
6–10	105
11–15	115
16–20	52
Total	329

### Common Method Variance

As this study data collection was from a single source, Podsakoff et al.’s (2003) guidelines were considered CMB. We excluded the titles of dimensions and constructs to lessen the causal effect of informants’ realizing what was being put to the test. Respondents’ confidentiality was also assured, and respondents were assured that there was no wrong or incorrect answer. Moreover, this study also applied full collinearity by following the guidelines of Kock (2015). Therefore, the single-source issue through full collinearity is addressed as all the values are well below 3 (Kock and Lynn, 2012), as shown in Table 2.

**TABLE 2:** Full collinearity testing.

GHRM	GKS	GC	GB
1.752	1.457	1.319	1.576

*GHRM, green human resource management; GKS, green knowledge sharing; GC, green commitment; and GB, green behavior.*

Moreover, this study employs SMART PLS 3 (Ringle et al., 2015; Hair et al., 2019) as this software does not need the assumption of data normality (Chin et al., 2003). By following the suggestions of Cain et al. (2017) and Hair et al. (2019), we assessed data normality by applying Mardia’s multivariate method of skewness (β = 2.770, *p* < 0.01) and kurtosis (β = 28.135804, *p* < 0.01). This was done through web power, and the results show multivariate non-normal data.

### Measurement Model

We applied the measurement model by considering the suggestions by Hair et al. (2019) and Islam et al. (2022). For the measurement model, we assessed the loadings, average variance extracted (AVE), and the composite reliability (CR). This study fulfills the minimum requirements of the needed values of composite reliability (CR > 0.7), average variance extracted (AVE > 0.5), and factor loadings (loadings > 0.5) for the purpose of establishing convergent validity in the measurement model as presented in Table 3.

**TABLE 3:** Measurement model convergent validity.

Latent variable	Items	Factor loadings	AVE	CR
GHRM	GHRM1	0.836	0.657	0.919
	GHRM2	0.879		
	GHRM3	0.863		
	GHRM4	0.693		
	GHRM5	0.774		
	GHRM6	0.802		
GKS	GKS1	0.848	0.655	0.938
	GKS2	0.877		
	GKS3	0.799		
	GKS4	0.864		
	GKS5	0.749		
	GKS6	0.859		
	GKS7	0.768		
	GKS8	0.692		
GC	GC1	0.836	0.626	0.930
	GC2	0.761		
	GC3	0.784		
	GC4	0.757		
	GC5	0.795		
	GC6	0.854		
	GC7	0.821		
	GC8	0.710		
GOB	GOB1	0.848	0.654	0.918
	GOB2	0.870		
	GOB3	0.759		
	GOB4	0.879		
	GOB5	0.804		
	GOB6	0.673		

In the next step, Henseler et al.’s (2015) and Franke and Sarstedt’s (2019) guidelines were employed to ensure discriminant validity by applying HTMT criteria. All the values are shown in Table 4, showing the values of all constructs less than 0.85.

**TABLE 4:** Discernment validity: HTMT criterion.

Constructs	GHRM	GKS	GC	GOB
GHRM				
GKS	0.618			
GC	0.380	0.180		
GOB	0.284	0.426	0.501	

### Hypotheses Testing

We followed Hair et al.’s (2019) recommendations by applying 5,000 re-sample bootstrapping procedures for testing hypotheses. We used *p*-values, confidence intervals, and effect sizes to conclude the significant results of the hypotheses as suggested by Hahn and Ang (2017; refer to Table 5). Our study found significant paths: GHRM was found to have a significant positive relationship with GC (β = 0.396, *t* = 5.830, *p* = 0.000, *f*^2^ = 0.133), and GC was found to have a significant positive relationship with GOB (β = 0.300, *t* = 5.992, *p* = 0.000, *f*^2^ = 0.122), hence hypotheses 1 and 2 are supported. To test for the mediation hypothesis (H3), this study applied a bootstrapping procedure with a resample of 5,000. The 95% bias-corrected bootstrap confidence interval of the indirect effect was generated to test the existence of the mediation effect of GC (Preacher and Hayes, 2008). In Table 5, the 95% bias-corrected bootstrap confidence interval values did not straddle a 0 in between, indicating the presence of mediation. Therefore, this study confirms and concludes that GHRM indirectly affects green behaviors through GC.

**TABLE 5:** Path coefficient.

	Relationship	β		CIBC	*t*-value	*p*-value	*f* ^2^	Decision
		5%	95%					
H1	GHRM → GC	0.396	0.266	0.520	5.830	0.000	0.133	Supported
H2	GC → GB	0.300	0.203	0.395	5.992	0.000	0.122	Supported
H3	GHRM → GC → GOB	0.110	0.074	0.183	3.690	0.000	–	Supported
H4	GHRM*GKS → GC	0.237	0.044	0.330	3.608	0.000	0.062	Supported
H5	GHRM*GKS → GC→GB	0.071	0.037	0.116	2.841	0.002	–	Supported

**FIGURE 2:**
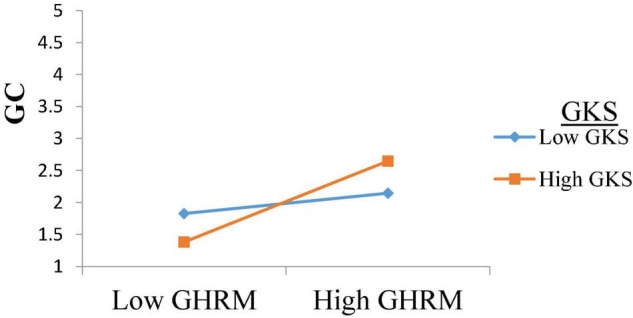
Interaction effect of GHRM and green knowledge sharing.

Furthermore, we tested the moderating role of GKS between GHRM and GC as per the suggestions by Hair et al. (2019). The results has shown that the interaction term of GHRM*GKS (β = 0.237, *t* = 3.608, *p* = 0.000, *f*^2^ = 0.062) is significant. The *f*^2^ effect size value of the interaction term (i.e., GHRM*GKS = 0.062) indicates a medium effect as per Kenny et al. (2016). Dawson (2014) suggestions are also followed to plot the significant interaction effect, as shown in Figure 2. The graph suggests that the relationship between GHRM and GC is stronger when GKS is high. Hence, H4 is also supported.

This study applied a two-stage approach as Hair et al. (2019) recommended in the Smart partial least square structural equation modeling to test the moderated mediation. Based on hypothesis H5, it was anticipated that GKS would moderate the relationship between GHRM and GC. Additionally, the conditional indirect effects or moderated mediation hypothesis is also supported. As evident from the results in Table 5, the indirect effect of GHRM on EGB through GC is conditional upon GKS (i.e., β = 0.071, *t* = 2.841, *p* = 0.002). This means that GKS moderates the indirect relationship between GHRM and EGB through GC such that this relationship is stronger in the presence of high GKS. Therefore, H5 is supported.

## Discussion

Extant literature has highlighted that factors effecting EGB are in their infancy stage and require more attention to explore different factors of the underlying mechanisms of GHRM and green behavior in the workplace (Yong et al., 2020b; Rubel et al., 2021; Islam et al., 2022). In the same vein, by employing social learning theory and attitude theory, this study investigated the role of GKS as a moderator of the indirect influence of GHRM on EGB *via* GC. The study findings lend support to the proposed moderated mediation model by finding that GKS moderates the indirect effect of GHRM on EGB through GC. Our study supports that GC mediates the relationship between GHRM and EGB because the results showed that perceived GHRM has a significant positive effect on perceived GC, and GC has a direct positive significant effect on EGB. Hence, the first three hypotheses of the study are substantiated, which supports Bull’s (1951) attitude theory. Attitude theory signifies the importance of employees’ positive behaviors like affective commitment (Babakus et al., 2003) in the mediation of employees’ positive evaluations of management practices and their positive responses. Based on attitude theory, our study supports that organizations adopting and implementing GHRM practices make employees more committed to their environment, which further affects their pro-environmental behavior in the workplace. Moreover, our study supported the moderating role of green knowledge between GHRM and GC. The study results show that the positive relationship between GHRM and GC is strengthened by the high level of GKS in the workplace. Hence, this study substantiates hypotheses 4 and 5 and hence aligns with social learning theory, which describes learning in the social context as a cognitive process that can be realized by instructions or observations even if there is no direct reinforcement (Bandura, 1977). It means that employees who adopt organizations’ GHRM initiatives and share green knowledge will also influence other members and can become a source of inspiration and can help the rest of the employees to adopt the same practices to become part of the members’ follow green practices in the working relationship. This consequently has a positive influence on employees’ learning with their environmental commitment and green performance. The findings of the study are consistent with previous studies on the role of GHRM in environmental management (Ren et al., 2018; Ansari et al., 2021; Islam et al., 2021a). Hence, by conducting this research, we justify and support how organizations may affect workers’ green behaviors as employees for sustainable organizations. This study has notable theoretical and practical contributions, which are discussed below.

### Theoretical Implication

The findings of our study have made manifold contributions to advancing theory. Attitude theory (Bull, 1951) and social learning theory have been used to explain the theoretical underpinnings of this study. Attitude theory (Bull, 1951) supports the mediating role of GC, while social learning theory supports GKS as a moderating variable. Attitude theory signifies the importance of employees’ positive behaviors like affective commitment (Babakus et al., 2003) in the mediation of employees’ positive evaluations of management practices and their positive responses. Findings support attitude theory because our study result shows that organizations adopting and implementing GHRM practices make employees more committed to their environment (Pham et al., 2019). Prominent scholars (Tuan, 2021) have proposed the importance of GC as a mediator linking GHRM to the various employee and organizational outcomes. Therefore, GC can be viewed as an employee commitment to environmental issues (Paillé et al., 2014), serving as an intermediary link between GHRM and green behavior. Therefore, an organization’s GHRM practices affect employees’ GC s, affecting employees’ green behaviors.

Furthermore, social learning theory states that individuals learn new behaviors by observing and imitating others. This theory describes learning in the social context as a cognitive process that can be realized by instructions or observations even if there is no direct reinforcement. In the support of social learning theory, our study results show that employees who adopt organizations’ GHRM initiatives and share green knowledge will also influence other members become a source of inspiration and can help the rest of the employees to adopt the same practices to become the part of the members’ follow green practices in the working relationship. This consequently has a positive influence on employees’ learning with their environmental commitment and green performance. Therefore, our study contributes to the literature by empirically testing the relationship of GHRM with employees’ green outcomes. We used the behavioral HRM (Nishii et al., 2008; Jackson, 2013; Kim et al., 2019) and EGB (Kramar, 2014; Dumont et al., 2017; Yusliza et al., 2017) literature to test the relationship between GHRM and EGB *via* GC. Furthermore, based on social learning theory, our study also contributes to the moderating role of knowledge sharing on the indirect influence of GHRM on green behavior through GC, as suggested by previous studies (Farrukh et al., 2019; Ansari et al., 2021). Therefore, organizations with GHRM practices encourage and enhance employee GC to influence green behaviors in organizations. Based on social learning theory and attitude theory, this study supports and explains that employees’ GKS behavior plays a moderating role in the indirect effect of GHRM on green behavior *via* GC. These results are consistent with previous studies (Pham et al., 2019; Rubel et al., 2021).

### Practical Implications

The green management perspective in organizations adopted in this study is crucial for organizational sustainability and environmental management. The practical implications of this study support firms’ employment of green practices as an instrumental and effective ways for their employees’ environment-friendly behaviors. Since our study found that GHRM and GKS can improve GC and green behavior, the management should therefore prioritize hiring and develop GKS behavior among managers and employees. The human resources department should communicate with employees about GHRM practices and their engagement in knowledge sharing behavior and share how such engagement is benefiting the business stakeholders. Furthermore, employees’ green behavior can be improved through the performance management system of the staff. For example, employees can be rewarded and promoted based on GKS behavior, GC, and green performance. When organizations invest in GHRM, they accept the efforts needed in green hiring, educating and creating awareness levels among employees, and encourage their staff to gain their commitment to exhibit green behavior for the sustainable environmental performance of organizations. This is why organizations should devise a strategy for GHRM and GKS implementation that would help individuals and organizations contribute to social sustainability. Top management of organizations should devise a strategy and mechanism for GHRM practices and play a significant role in organizations’ environmental management. As found in this study, by considering GHRM and GKS, organizations create GC among employees and are more likely to influence their green behaviors for the sustainable performance of organizations.

### Limitations and Future Research Recommendations

Although current research provides theoretical and practical implications, this research was still not spared from its limitations. First, the employment of cross-sectional research does not establish causality among the constructs of this study. Future research may consider panel data to tap the causality of the same constructs to navigate the same problem. Second, this study has considered GC as a one-dimensional construct. Future research may explore the multidimensional perspective of GC. Third, this study has employed a quantitative method; future research may employ a sequential exploratory study to explore environmental management factors to triangulate the results. Moreover, future research may extend our model by including different personality types as moderating variables. Constructs like internal and external locus of control, big-personality models, and other similar models may be tested in relation to green service behavior. Finally, future studies may extend GHRM to empirically test green ability, green motivation, and green opportunity relationships through the lens of AMO theory.

## Conclusion

We inferred in our study that the role of GHRM is of crucial importance for organization sustainability, while acceptance of study hypotheses shows the significance of GHRM in bringing desired green behaviors through GKS and GC. Green knowledge sharing moderates the indirect influence of GHRM on green behavior *via* GC. Therefore, GHRM, GKS, and GC are essential for employees and organizations. Moreover, the limitations and implications of our study provide an opportunity for future research in the same domain.

## Data Availability Statement

The original contributions presented in this study are included in the article/supplementary material; further inquiries can be directed to the corresponding author.

## Ethics Statement

Ethical review and approval was not required for the study on human participants in accordance with the local legislation and institutional requirements. The patients/participants provided their written informed consent to participate in this study.

## Author Contributions

KK has taken the overall responsibility for the manuscript and gave the idea of the issue to be investigated. MS has written the Introduction Part. QK worked on Literature Review section of the manuscript and has taken the responsibility of data collection. SA helped in the methodology part and ran the statistical analysis. MN has compiled the discussion part and he has provided technical support throughout the manuscript. All authors contributed to the article and approved the submitted version.

## Conflict of Interest

The authors declare that the research was conducted in the absence of any commercial or financial relationships that could be construed as a potential conflict of interest.

## Publisher’s Note

All claims expressed in this article are solely those of the authors and do not necessarily represent those of their affiliated organizations, or those of the publisher, the editors and the reviewers. Any product that may be evaluated in this article, or claim that may be made by its manufacturer, is not guaranteed or endorsed by the publisher.
